# Role of dynamic ctDNA monitoring in cervical and anal epidermoid carcinomas under curative chemoradiation

**DOI:** 10.1016/j.clinsp.2026.101048

**Published:** 2026-07-14

**Authors:** Camila M.Venchiarutti Moniz, Renata Colombo Bonadio, Felippe Lazar, Andre Tsin Chih Chen, Renan Ribeiro e Ribeiro, Mariana Siqueira, Maria Ignez Braghiroli, Marcela Crosara, Cinthia Ortega, Caio Nahas, Karim Yaqub Ibrahim, Lucila Rocha, Abraão Dornellas, Vanessa da Costa Miranda, Evandro Sobroza de Mello, Daniela Ribeiro Nebuloni Nagy, Maria Luiza Nogueira Dias Genta, Mariana de Paiva Batista, Milena Giulia Gonçalves, Laura Sichero, Jorge Sabbaga, Carlos Frederico Sparapan Marques, Carolina Ribeiro Victor, Maria Del Pilar Estevez-Diz, Paulo M. Hoff

**Affiliations:** a Instituto D’Or de Pesquisa e Ensino (IDOR), São Paulo, SP, Brazil; b Instituto do Câncer do Estado de São Paulo (ICESP), São Paulo, SP, Brazil; c Instituto D’Or de Pesquisa e Ensino (IDOR), Rio de Janeiro, RJ, Brazil; d Instituto D’Or de Pesquisa e Ensino (IDOR), Brasília, DF, Brazil; e Comprehensive Center for Precision Oncology, Universidade de São Paulo, São Paulo, SP, Brazil; f Universidade de São Paulo, São Paulo, SP, Brazil

**Keywords:** ctDNA monitoring, Cervical epidermoid carcinoma, Anal epidermoid carcinoma, Curative chemoradiation

## Abstract

**Background:**

Circulating tumor DNA (ctDNA) is a promising biomarker to predict recurrence in solid tumors such as colorectal and breast cancer. Defining complete response after chemoradiation remains challenging, as post-treatment inflammatory and tissue changes limit clinical and imaging evaluation.

**Methods:**

The authors conducted a prospective longitudinal study of patients with localized cervical and anal cancer treated with curative-intent chemoradiation with the aim of evaluating the role of ctDNA as a predictor of recurrence. Blood specimens were collected at D1, D29, immediately post-treatment, 8-weeks, 24-weeks, and every 6-months up to 5-years. ctDNA testing (Signatera™) was provided by Natera as an in-kind donation. The primary endpoint was correlation between 8-week ctDNA and recurrence.

**Results:**

28 patients were evaluable. Ten recurrences occurred, all in patients with positive ctDNA after chemoradiation and before radiologic progression. The median interval between ctDNA positivity and radiologic progression was 3.0-months (range 0.5–21.3). All patients with radiologic complete response at 6 months had negative 8-week ctDNA. Among those without complete response, 8-week ctDNA identified most who later progressed (6/8), while all without complete response who did not progress had negative ctDNA. Eight-week ctDNA positivity was strongly associated with inferior progression-free survival: median 6.7-months in ctDNA-positive versus not reached in ctDNA-negative patients (HR = 40.09, p = 0.001).

**Conclusion:**

Among patients with cervical and anal cancer, ctDNA monitoring after definitive chemoradiation identifies individuals without complete response and predicts recurrence. ctDNA positivity at 8-weeks identifies a very high-risk group for whom treatment intensification trials are warranted.

## Highlights

• ctDNA predicts recurrence after chemoradiation in cervical and anal cancer.
• 8-week post-CRT ctDNA positivity yields 100% PPV for disease recurrence.
• ctDNA detects relapse a median of 3 months before radiologic progression.
• Negative ctDNA correlates with radiologic complete response at 6-months.
• ctDNA may guide surveillance and treatment escalation in HPV cancers.

## Introduction

HPV-related cancers, including cervical and anal cancer Squamous Cell Carcinoma (SCC), remain a significant health problem. According to GLOBOCAN 2022 data, there were 662.301 new cases and 348.874 deaths from cervical cancer worldwide, with approximately 90% of new cases and 94% of deaths occurring in low- and middle-income countries.^1^^,^^2^ According to the most recent global estimates, approximately 50,685 new cases of anal cancer and 19,293 related deaths were reported worldwide up to 2022.^1^^,^^2^

Definitive chemoradiation is the standard-of-care approach for oncologic treatment in advanced localized disease. However, this strategy will cure around 60% of anal and cervical cancer, considering T4 and node-positive populations.^3^^,^^4^ Patients who are not complete responders to this strategy must be rapidly identified to have a chance of a surgical rescue with curative intent. Therefore, in clinical practice, the acute inflammation during chemoradiation and the post-treatment fibrosis process becomes difficult to access and early define non-responders with residual disease using conventional clinical and image evaluation, with some studies showing late image responders.^5^ The first image evaluation is recommended by current guidelines 8‒12 weeks until 26-weeks after chemoradiation is completed.^6^

In this scenario, the circulating tumor DNA (ctDNA) assessment emerges as a promising tool for early monitoring of residual disease. The ctDNA is a fraction of cell-free circulating DNA derived from tumor cells. This biomarker has proven helpful in specific colorectal and breast cancer scenarios to improve real-time monitoring of disease evolution, therapy response, and prognosis prediction.^7^^,^^8^

The authors propose a prospective study to evaluate the role of ctDNA dynamic monitoring in a cohort of patients treated with standard-of-care definitive chemoradiation. The primary hypothesis was that ctDNA status at 8 weeks post-chemoradiation would correlate with progression-free survival, enabling earlier identification of patients at risk of disease recurrence than conventional radiologic follow-up. Secondary objectives included evaluation of ctDNA kinetics throughout follow-up, correlation of ctDNA positivity with radiologic response, and assessment of the lead time between ctDNA detection and radiologic disease progression.

## Patients and methods

This study is a subgroup evaluation of a prospective multicentric cohort of anal and cervical cancer “Real-World Data in HPV-Related Anogenital Tumors”. In the present analyses, the authors included patients with localized disease between 2020 and 2023 treated at Instituto do Câncer do Estado de São Paulo - ICESP and at Oncologia D'OR/Instituto D'Or de Pesquisa e Ensino. The sample size for this analysis was estimated at 30 patients by convenience. The criteria for inclusion in this subgroup analysis were patients with previous histological diagnosis of squamous cell carcinoma of the anal canal or squamous cell carcinoma cervical cancer, age > 18 years, and candidates for standard-of-care chemoradiation with curative treatment. Stage was reported using AJCC VIII Edition for anal cancer and FIGO for cervical cancer. The study was approved by the Local Ethics Committee. All patients provided informed consent for participating in this study.

Blood samples were collected at predefined time points during chemoradiotherapy for ctDNA analysis: prior to treatment initiation, at day-29 (midpoint of chemoradiation), one week after completion of chemoradiation, at 8- and 24-weeks post-chemoradiation, every six months during the first 1–2 years of follow-up, and annually from years 3 to 5 of follow-up (Supplementary Fig. 1). Tumor tissue specimens collected before treatment initiation were Formalin-Fixed and Paraffin-Embedded (FFPE) and subsequently analyzed in parallel with serial blood samples for ctDNA monitoring using Signatera™, a personalized and tumor-informed multiplex PCR-based assay (bespoke multiplex polymerase chain reaction Next-Generation Sequencing ctDNA assay, Natera). ctDNA testing (Signatera™) was provided by Natera as an in-kind donation.

The primary endpoint was the correlation with ctDNA levels 8-weeks after chemoradiation with progression-free survival. Secondary endpoints included the correlation of ctDNA levels with 24-week image, correlation of ctDNA detection during any time point of follow-up with risk of recurrence, interval between ctDNA detection and radiologic progression, and Overall Survival (OS) according to ctDNA levels at 8-weeks. Radiologic response was assessed using pelvic Magnetic Resonance Imaging (MRI) or computed tomography (if MRI contraindicated), and thorax and abdomen computed tomography, at 8–12 weeks and at 6-months post-chemoradiation. Response classification followed standard clinical criteria (complete response, partial response, stable disease, or progressive disease). The 6-month post-chemoradiation was used as the standard for radiologic evaluation of response, as late responses may occur after curative chemoradiation for anogenital tumors. Radiologic assessments were performed by institutional radiologists as part of routine clinical care; independent centralized or blinded radiologic review was not performed in this analysis, which represents a methodological limitation. Diagnostic accuracy was evaluated using sensitivity, specificity, Positive Predictive Value (PPV), and Negative Predictive Value (NPV), with 95% confidence intervals calculated using the Wilson score method. Cervical and anal cancer patients were analyzed jointly given that both are HPV-associated squamous cell carcinomas sharing the primary clinical challenge of early identification of residual disease after definitive chemoradiation. Subgroup analyses by tumor type are presented where sample sizes permit, and should be interpreted as exploratory given the small numbers in each subgroup.

Progression-free survival was calculated from the date of chemoradiation initiation until the date of disease progression, recurrence, or death from any cause. Overall survival was calculated from the date of chemoradiation initiation until the date of death from any cause. Patients without these events were censored at the date of last follow-up. Survival estimates were performed using the Kaplan-Meier method, with the log-rank test to compare the difference between survival curves. Cox regression was used to estimate the hazard ratio (HR) and 95% confidence interval. Given the small number of events, all hazard ratio estimates should be considered exploratory and interpreted with caution. Cox regression for overall survival was not performed due to an insufficient number of events to yield a reliable estimate. Statistical analyses were performed using the R and Stata software (version 15.0, StataCorp, Texas, USA); p-values lower than 0.05 were considered statistically significant.

The study was reviewed and approved by the Research Ethics Committee of Hospital e Maternidade São Luiz (CAAE 17901719.4.2002.0087).

Written informed consent was obtained from all participants prior to study inclusion. Before enrollment, eligible patients received detailed verbal and written information about the study objectives, procedures, potential risks and benefits, and data confidentiality. Participants were given sufficient time to ask questions and to decide on participation, and consent was obtained voluntarily before any study-specific procedures were initiated.

The manuscript is in accordance with STARD guidelines for diagnostic and prognostic studies. In accordance with the journal’s guidelines, the authors will provide the present data for independent analysis by a selected team of the Editorial Team for the purposes of additional data analysis or for the reproducibility of this study in other centers if such is requested.

## Results

Between 2020 and 2023, 33 patients were included. In terms of patients’ characteristics, four were excluded due to insufficient material to run the Signatera™ test, and one did not start chemoradiation due to disease progression and clinical deterioration. Twenty-eight patients were evaluable for ctDNA monitoring; 15 had anal cancer and 13 cervical cancers. Table 1 illustrates baseline characteristics according to the study cohort. In both cohorts, most patients had locally advanced disease – 73.3% were stage III in the anal cancer cohort and 61.6% stage III‒IVA in the cervical cancer cohort. All patients received CT concomitant with radiotherapy, with 100% receiving cisplatin in cervical cancer, and 80% cisplatin plus capecitabine in the anal cancer group. HIV serology was positive in 3 patients in the anal cancer cohort. Among patients with HPV status available, 90% (n = 18/20) were HPV-positive; six of them (30%) had more than one type of HPV detected.

**Table 1:** Patients’ characteristics and treatment received according to primary tumor site.

Variable	Category	n	%
**Anal Cancer (n = 15)**			
Sex	Female	11	(73.3)
	Male	4	(26.7)
Age, median (range)	62 (48–86)		
T stage	T1	1	(6.7)
	T2	5	(33.3)
	T3	6	(40.0)
	T4	3	(20.0)
N stage	N0	5	(33.3)
	N1a	3	(20.0)
	N1b	4	(26.7)
	N1c	2	(13.3)
	Nx	1	(6.7)
M stage	M0	15	(100.0)
Stage (AJCC VIII)	Stage I	1	(6.7)
	Stage II	3	(20.0)
	Stage III	11	(73.3)
Concomitant Chemotherapy	Capecitabine + Cisplatin	12	(80.0)
	5-FU + Cisplatin	1	(6.7)
	5-FU + Mitomycin	1	(6.7)
	5-FU Monotherapy	1	(6.7)
HIV Status	Positive	3	(20.0)
	Negative	9	(60.0)
	Unknown	3	(20.0)
HPV Status	Positive	10	(66.7)
	HPV 11	2	(13.3)
	HPV 16	8	(53.3)
	HPV 45	1	(6.7)
	HPV 58	2	(13.3)
	Negative	0	(0.0)
	Unknown	5	(33.3)
**Cervical Cancer (n = 13)**			
Sex	Female	13	(100.0)
Age, median (range)	42 (25–71)		
T stage	T1	2	(15.4)
	T2	5	(38.5)
	T3	3	(23.1)
	T4	3	(23.1)
N stage	N0	8	(66.7)
	N1	4	(33.3)
M stage	M0	12	(92.3)
	Mx	1	(7.7)
Stage (FIGO)	Stage I	1	(7.7)
	Stage II	4	(30.8)
	Stage III	5	(38.5)
	Stage IVA	3	(23.1)
Concomitant Chemotherapy	Cisplatin	13	(100.0)
HIV Status	Positive	0	(0.0)
	Negative	9	(69.2)
	Unknown	4	(30.8)
HPV Status	Positive	7	(53.8)
	HPV 11	2	(15.4)
	HPV 16	5	(38.5)
	HPV 45	1	(7.7)
	HPV 51	4	(30.8)
	Negative	2	(15.4)
	Unknown	4	(30.8)

AJCC, American Joint Committee on Cancer; FIGO, International Federation of Gynecology and Obstetrics; 5-FU, 5-Fluorouracil; HIV, Human Immunodeficiency Virus; HPV, Human Papillomavirus.

* HPV subtypes are listed for HPV-positive patients only. Some patients had more than one HPV type detected; therefore, subtype counts may exceed the total HPV-positive count.

One patient in the cervical cancer cohort received neoadjuvant therapy before definitive chemoradiation, and the first ctDNA before treatment was used as a baseline pretreatment. Baseline ctDNA was detectable in all 28 evaluable patients (100%).

Ten patients with anal cancer and eleven with cervical cancer underwent radiologic evaluation at 6-months. Regarding the correlation of ctDNA and radiologic response, at this time point, 40% (4/10) of the anal cancer patients and 27.3% (3/11) of the cervical cancer patients had achieved a radiologic complete response. All individuals with a radiologic complete response had negative circulating tumor DNA (ctDNA) at 8-weeks post-chemoradiation.

Among the 14 patients without radiologic complete response at 6-months, 8 developed disease progression, while six did not. All six patients without progression had negative ctDNA at 8 weeks. Of the eight with progression, six had positive ctDNA at 8-weeks, and the remaining two developed detectable ctDNA later during follow-up. These findings suggest that, in patients without a radiologic complete response at 6-months, ctDNA status at 8-weeks and throughout follow-up predict risk of disease progression.

**Fig. 1:**
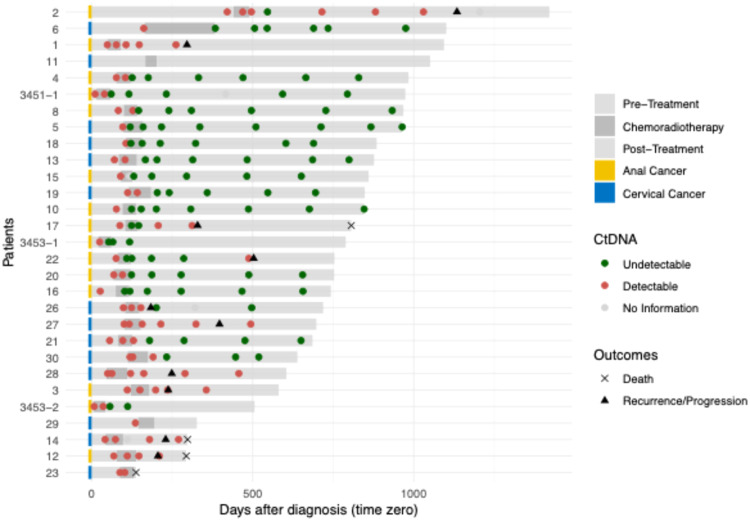
Swimmer plot illustrating the temporal evolution of ctDNA status, disease recurrence/progression, and death for each patient. Each horizontal bar represents one patient; bar length represents duration of follow-up from the date of diagnosis (x-axis, months). Bars are color-coded by disease status: blue bars represent patients with cervical cancer; yellow bars represent patients with anal cancer. Circles along each bar indicate ctDNA assessments: green circles represent negative (undetectable) ctDNA and red circles represent positive (detectable) ctDNA. Triangles indicate the timing of radiologic disease recurrence or progression. An “X” symbol indicates death. Patients are ordered from longest to shortest follow-up.

After a median follow-up of 23-months, 10 recurrences and one death without recurrence were observed. Analysis of ctDNA dynamics in relation to radiologic progression is detailed in Supplementary Table 1 presents the ctDNA levels for each patient at each timepoint. Fig. 1 illustrates the ctDNA dynamic monitoring at each timepoint and the moment of radiologic disease progression/ recurrence and death.

Among the ten participants who experienced radiological disease progression, all had a positive ctDNA level in at least one timepoint after conclusion of chemoradiation prior to radiologic progression. One of them had a positive ctDNA soon after chemoradiation completion and had a radiologic progression before the 8-week landmark. Between the 9 others, seven had a positive ctDNA at 8 weeks, and two had a negative ctDNA at 8-weeks that later became positive during sequential monitoring.

Considering the 8-week ctDNA assessment, all seven patients with positive ctDNA at 8-weeks experienced disease recurrence, resulting in a PPV of 100% (95% CI 64%–100%). Among the 19-patients with negative ctDNA at 8-weeks, 3 developed disease progression/recurrence during follow-up, and 16 remained recurrence-free, corresponding to an NPV of 84.2% (95% CI 60%–97%). The sensitivity and specificity of the 8-week ctDNA test were 70% (95% CI 35%–93%) and 100% (95% CI 79%–100%), respectively. In comparison, the 6-month radiologic response showed a sensitivity of 100% (all patients who recurred failed to achieve a complete radiologic response), but a specificity of only 53.8% (many patients without recurrence also failed to achieve a complete radiologic response at 6-months). Overall, 8-week ctDNA had a higher accuracy than 6-month radiologic response (88.5%vs. 71.4%). The 8- to 12-week radiologic response was also assessed in an exploratory manner, yielding an accuracy of 69%. Table 2 summarizes the diagnostic accuracy metrics, including sensitivity, specificity, PPV, and NPV, with corresponding 95% confidence intervals, for 8-week ctDNA, 6-month radiologic response, and 8- to 12-week radiologic response.

**Table 2:** Diagnostic performance of 8-week ctDNA and 6-month radiologic response as predictors of disease recurrence in anogenital cancer after definitive chemoradiation.

Test	Sensitivity	Specificity	PPV	NPV	Accuracy
8-week ctDNA, % (95% CI)	70% (40–89%)	100% (81–100%)	100% (64–100%)	84.2% (62–94%)	88.5% (71–96%)
8- to 12-week radiologic response, % (95% CI)	90% (59–98%)	53.8% (29–77%)	60% (36–80%)	87% (52–98%)	69.6% (49–84%)
6-month radiologic response, % (95% CI)	100% (67–100%)	53.8% (29–77%)	57.1% (33–79%)	100% (65–100%)	71.4% (50–86%)

ctDNA, circulating tumor DNA; PPV, Positive Predictive Value; NPV, Negative Predictive Value.

These results remained similar in a subgroup exploratory analysis according to tumor type. For anal cancer, the PPV, NPV, sensitivity and specificity were 100% (95% CI 51%–100%; n = 4/4), 81.8% (95% CI 52%–95%; n = 9/11), 66.7% (95% CI 30%–90%; n = 4/6), and 100% (95% CI 70%–100%; n = 9/9). Of note, among patients with anal cancer, no difference was observed between patients with or without a recurrence regarding the type of concurrent chemotherapy received (p = 1.000). All patients with a recurrence had received concurrent capecitabine plus cisplatin, which was the most used regimen in the cohort. For cervical cancer, the PPV, NPV, sensitivity and specificity were 100% (95% CI 44%–100%; n = 3/3), 87.5% (95% CI 53%–98%; n = 7/8), 75% (95% CI 30%–95%; n = 3/4), and 100% (95% CI 65%–100%; n = 7/7) (Supplementary Table 2).

**Fig. 2:**
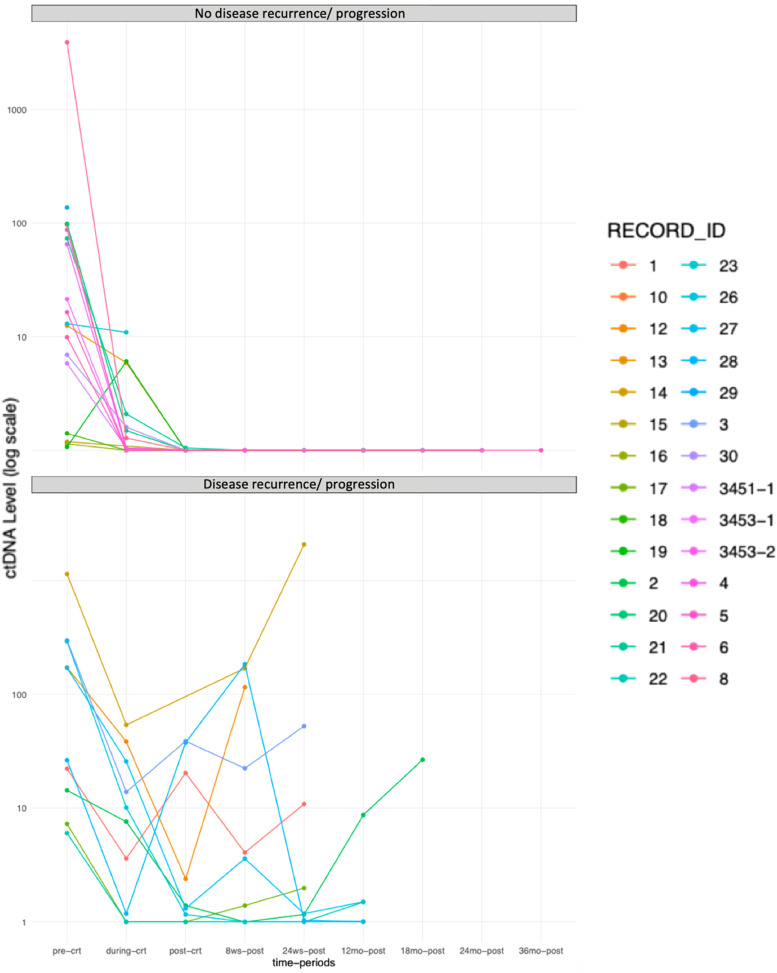
Spider plot of ctDNA levels over time among patients with cervical or anal cancer treated with definitive chemoradiation, stratified by disease recurrence/progression status. Each colored line represents one patient’s serial ctDNA measurements. The y-axis represents the ctDNA level (mean tumor molecules/mL, log scale) and the x-axis represents the timepoint of ctDNA collection. Trajectory endpoints reflect the last available ctDNA measurement for each patient, corresponding to the collection prior to the last follow-up visit (censored patients), date of disease progression, or death. ctDNA, Circulating tumor DNA; pre-crt, pre chemoradiation; during-crt, During chemoradiation; post-crt, Post chemoradiation; 8ws-post, 8-weeks post chemoradiation; 24ws-post, 24-weeks post chemoradiation; 12mo-post, 12-months post chemoradiation; 18mo-post, 18-months post chemoradiation; 24mo-post, 24-months post chemoradiation; 26mo-post, 36-months post chemoradiation.

In the spider plot (Fig. 2), the ctDNA dynamics are illustrated for the groups with and without disease recurrence/ progression. Patients with no disease progression usually have an early and sustained clearance of ctDNA, while those with disease progression exhibited a more irregular pattern, with the majority maintaining elevated ctDNA levels during treatment. Three pts had undetectable ctDNA levels during the initial follow-up, followed by detectable ctDNA in subsequent assessments, preceding radiologic progression. The median time from a positive post-treatment ctDNA level and a radiologic progression was 3.0-months (range 0.5–21.3 months).

**Fig. 3:**
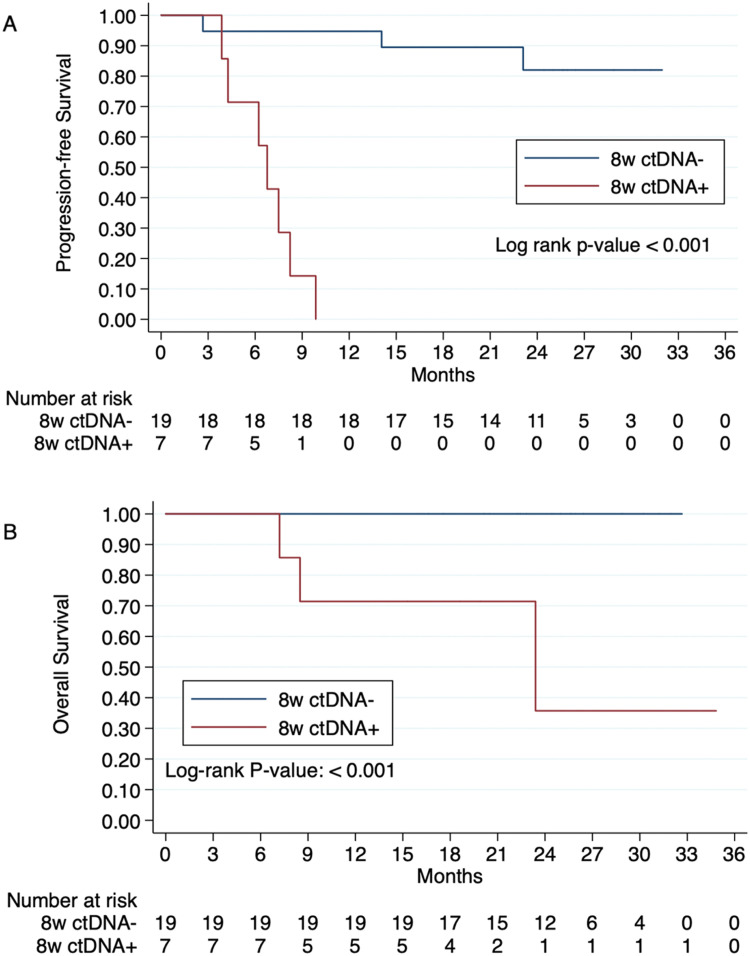
Progression-free survival and overall survival of patients with cervical and anal cancer after definitive chemoradiation according to 8-week ctDNA status. 8 w, 8-weeks; ctDNA+, Detectable ctDNA; ctDNA-, undetectable ctDNA.

Patients’ progression-free and overall survival according to ctDNA status at 8-weeks after chemoradiation showed marked differences between groups. Patients with detectable ctDNA at 8-weeks after chemoradiation had a substantially higher risk of disease recurrence, progression, or death. Median progression-free survival was 6.7 months in the ctDNA-positive group and was not reached in ctDNA-negative patients (HR = 40.09, 95% CI 4.62–347.35, p = 0.001). The 2-year progression-free survival rates were 0% and 82%, respectively (Fig. 3). In an exploratory subgroup analysis according to tumor type, patients with positive 8-week ctDNA had a median progression-free survival of 4.3-months in the anal cancer cohort and 6.8-months in the cervical cancer cohort, while median progression-free survival was not reached in ctDNA-negative patients in either cohort (Supplementary Fig. 2). Regarding the site of recurrence, 6 patients (60%) had locoregional recurrence, 2 (20%) distant recurrence, and 2 (20%) both, with no distinction in site of recurrence according to 8-week ctDNA level (p = 1.000).

During the same period, four deaths were recorded, with a median overall survival of 23.4 months in the 8-week ctDNA-positive group and not reached in the 8-week ctDNA-negative group. Two-year overall survival rates were 35.7% and 100%, respectively (Fig. 3). A formal Cox regression for overall survival was not performed due to the low number of events (n = 4 deaths), which precluded a reliable hazard ratio estimate. These OS data are presented in descriptive terms only.

## Discussion

### Summary of main results

Surveillance after definitive treatment is a challenge in anogenital tumors. For anal cancer stages II and III, NCCN guidelines recommend at least an annual CT scan and/or MRI for three years. Clinical evaluation includes digital rectal and lymph node exams every 3–6 months for five years, plus anoscopy every 6–12 months for three years. However, image and clinical results are complicated to interpret due to the possibility of inflammation post-chemoradiation, slow tumor regression, and/or focus on active residual disease.^9^ In persistent or recurrent disease, salvage surgery with abdominoperineal resection can provide locoregional control in up to 77% of patients; however, the morbidity is high, and precise identification of the patients that will benefit from this procedure is essential.^10^ ctDNA/HPV ctDNA emerges as a tool for early detection of treatment failure with a minimally invasive test in breast, bladder, and oropharyngeal cancer.^11^^,^^9^ In anogenital tumors, considering the high HPV prevalence in these cases, available studies explored the monitoring of circulating HPV by droplet-digital PCR. In previous literature, it was detected in 63%–94% of cervical cancer patients with locally advanced cervical cancer.^13^ The present cohort's personalized tumor-specific assay test, with targets per patient, could explain the high ctDNA+ pre-chemoradiation rates (100%). Furthermore, the studied population has high-risk clinical features for recurrence, including advanced T and N status.

### Results in the context of published literature

The persistence of ctDNA or ctDNA HPV after first-line or definitive therapy can predict cervical and anal cancer relapse in other reports.^9^^,^^12^^,^^13^ The sensitivity for cHPV DNA ranged from 6%–100% for cervical cancer and 91%–100% for anal carcinoma. In addition, a relevant association was seen between the incidence and levels of cHPV DNA and the presence of metastasis or poor clinical outcomes in patients with non-metastatic cervical tumors.^13^ This study presented 70% (95% CI 40%–89%) sensitivity and 100% (95% CI 81%–100%) specificity at the 8-week timepoint, probably due to the advanced method to access ctDNA, with multiplex polymerase chain reaction Next-Generation Sequencing. None of the included studies in the meta-analysis was designed as a prospective clinical trial, and evaluation was not adjusted for the disease’s stage and severity or treatment modalities.^13^ Retrospective studies also do not determine specific time points for ctDNA collection, imaging, and clinical evaluation, and it is fundamental to guide the best time assessments to guide interventions and clinical decisions. Azzi et al. performed a ctDNA test in 251 patients (817 plasma samples) with stages I–IV of anal carcinoma. Thirty-seven patients had complete clinical information available with a median follow-up of 21-months. Among 27 patients analyzed with stages II and III, anytime ctDNA positivity post-definitive therapy was associated with a significantly shorter disease-free survival, with a median of 11.4-months in the ctDNA-positive group and not reached in the ctDNA-negative group (HR = 28.0, 95% CI 2.8–285.0; p = 0.005).^12^ The recently published phase III CALLA trial randomized 770 women with Locally Advanced Cervical Cancer (LACC) to receive durvalumab plus chemoradiation or chemoradiation alone. In this study, circulating tumor DNA (ctDNA) was assessed at baseline (detected in 98.9%), at Cycle 3 Day-1 (post-chemoradiation; detected in 35.5%), and at Cycle 6 Day-1 (three months post-chemoradiation; detected in 23.4%) in the durvalumab + chemoradiation arm, and in 39.8% and 36.4% of patients, respectively, in the chemoradiation-only arm. Consistent with the present results, clearance of ctDNA following chemoradiation was significantly associated with improved progression-free survival and Overall Survival (OS). Furthermore, patients with persistent ctDNA detection after chemoradiation exhibited an increased risk of disease progression, irrespective of treatment arm. Of note, HPV ctDNA was also evaluated in the trial, and although HPV ctDNA levels remain detectable in many cases, a higher decrease in HPV ctDNA levels also correlated with improved oncologic outcomes.^14^ It is important to note that 8–12-week imaging remains the current standard of care for post-treatment response assessment, and ctDNA should be considered a complementary biomarker rather than a replacement for radiologic evaluation. Indeed, in the exploratory analysis, 8–12 week imaging demonstrated sensitivity of 90% and NPV of 87%, suggesting an additive rather than superior role for ctDNA in this setting.

### Strengths and weaknesses

The present cohort’s personalized tumor-specific assay test, with targets per patient, could explain the high ctDNA+ pre-chemoradiation rates (100%). Furthermore, the studied population has high-risk clinical features for recurrence, including advanced T and N status. Several limitations of this study must be acknowledged. First, the sample size is small (n = 28 evaluable patients) with only 10 recurrence events, rendering all survival analyses ‒ including the PFS hazard ratio ‒ statistically unstable and exploratory in nature. The wide confidence intervals around both the diagnostic accuracy estimates and PFS HR (4.62–347.35) reflect this instability; no reliable Cox regression estimate could be obtained for overall survival given only four deaths. Second, the cohort pools two oncologically distinct diseases (cervical and anal cancer) that differ in staging systems, chemotherapy regimens, and patterns of failure; while both are HPV-associated squamous cell carcinomas sharing the clinical challenge of post-chemoradiation residual disease assessment, tumor-type-specific conclusions must be drawn cautiously from these small subgroups. Third, the anal cancer subgroup received heterogeneous chemotherapy regimens, which may have influenced ctDNA kinetics independently of tumor biology. Fourth, the comparison between 8-week ctDNA performance and 6-month radiologic response is limited by the different time points of assessment and the fact that radiologic progression partly defines the recurrence endpoint, introducing circularity. Fifth, radiologic response was assessed as part of routine clinical care without a protocol-mandated imaging modality or independent blinded radiologic review. Sixth, the median follow-up of 23-months is relatively short for the capture of late recurrences, particularly in cervical cancer. Finally, ctDNA testing was performed using a single tumor-informed assay (Signatera™), and results may not be generalizable to other ctDNA platforms. Despite these limitations, the present findings provide valuable hypothesis-generating data on the use of personalized ctDNA monitoring in anogenital tumors under chemoradiation, and form the scientific basis for prospective validation trials. Importantly, a false-negative rate of approximately 16% (3/19 patients with negative 8-week ctDNA who later recurred) indicates that ctDNA negativity alone cannot be used to guarantee disease-free status at this time.

### Implications for practice and future research

Based on these findings, the authors designed the ANA trial, currently recruiting patients (NCT06640283). This study aims to validate the use of ctDNA and HPV-ctDNA for surveillance and to guide treatment escalation in the ctDNA+ group. Patients who present with detectable ctDNA after chemoradiation without radiologic progression will receive complementary adjuvant treatment with an immune checkpoint inhibitor.

## Conclusion

In this hypothesis-generating, prospective cohort study, ctDNA dynamic monitoring in patients with cervical and anal tumors identified patients without complete response after definitive chemoradiation, with ctDNA at 8-weeks demonstrating a positive predictive value of 100% for disease recurrence. The current data provide a rationale for evaluating, in prospective trials, adjuvant treatment intensification approaches in ctDNA-positive patients. The ongoing ANA trial (NCT06640283) is designed to prospectively address this question. However, the observed 16% false-negative rate precludes any consideration of reduced surveillance in ctDNA-negative patients.

## Declaration of generative AI and AI-assisted technologies in the writing process

The authors used Large Language Models to improve the readability and language of the manuscript. After using this tool, the authors reviewed and edited the content as needed.

## Funding statement

All ctDNA assays (Signatera™) were provided by Natera as an in-kind donation for this study. This research did not receive any specific grant from funding agencies in the public, commercial, or not-for-profit sectors.

## CRediT authorship contribution statement

**Camila M.Venchiarutti Moniz:** Writing – original draft, Validation, Supervision, Project administration, Methodology, Formal analysis, Data curation, Conceptualization. **Renata Colombo Bonadio:** Writing – original draft, Validation, Supervision, Project administration, Methodology, Formal analysis, Data curation, Conceptualization. **Felippe Lazar:** Writing – review & editing, Methodology, Formal analysis, Data curation. **Andre Tsin Chih Chen:** Writing – review & editing, Methodology. **Renan Ribeiro e Ribeiro:** Writing – review & editing, Methodology. **Mariana Siqueira:** Writing – review & editing, Methodology. **Maria Ignez Braghiroli:** Writing – review & editing, Methodology. **Marcela Crosara:** Writing – review & editing, Methodology. **Cinthia Ortega:** Writing – review & editing, Methodology. **Caio Nahas:** Writing – review & editing, Methodology. **Karim Yaqub Ibrahim:** Writing – review & editing, Methodology. **Lucila Rocha:** Writing – review & editing, Methodology. **Abraão Dornellas:** Writing – review & editing, Methodology. **Vanessa da Costa Miranda:** Writing – review & editing, Methodology. **Evandro Sobroza de Mello:** Writing – review & editing, Methodology. **Daniela Ribeiro Nebuloni Nagy:** Writing – review & editing, Methodology. **Maria Luiza Nogueira Dias Genta:** Writing – review & editing, Methodology. **Mariana de Paiva Batista:** Writing – review & editing, Methodology. **Milena Giulia Gonçalves:** Writing – review & editing, Methodology. **Laura Sichero:** Writing – review & editing, Methodology. **Jorge Sabbaga:** Writing – review & editing, Methodology. **Carlos Frederico Sparapan Marques:** Writing – review & editing, Methodology. **Carolina Ribeiro Victor:** Writing – review & editing, Methodology. **Maria Del Pilar Estevez-Diz:** Writing – review & editing, Methodology. **Paulo M. Hoff:** Writing – review & editing, Validation, Supervision, Project administration, Methodology, Data curation, Conceptualization.

## Declaration of competing interest

Camila M Venchiarutti Moniz reports equipment, drugs, or supplies were provided by Natera. Camila M Venchiarutti Moniz reports a relationship with Nestle Health Science that includes: funding grants. Camila M Venchiarutti Moniz reports a relationship with Natera, Inc. that includes: funding grants. Camila M Venchiarutti Moniz reports a relationship with Libbs Pharmaceutical that includes: funding grants and speaking and lecture fees. Camila M Venchiarutti Moniz reports a relationship with AstraZeneca that includes: funding grants and speaking and lecture fees. Camila M Venchiarutti Moniz reports a relationship with Merck Sharp & Dohme Corp that includes: funding grants and speaking and lecture fees. Camila M Venchiarutti Moniz reports a relationship with Servier that includes: funding grants. Camila M Venchiarutti Moniz reports a relationship with Bristol-Myers Squibb Company that includes: funding grants. Camila M Venchiarutti Moniz reports a relationship with Pfizer that includes: funding grants. Camila M Venchiarutti Moniz reports a relationship with AbbVie Inc that includes: funding grants. Camila M Venchiarutti Moniz reports a relationship with Daiichi Sankyo Inc that includes: speaking and lecture fees. Camila M Venchiarutti Moniz reports a relationship with Roche that includes: funding grants. Camila M Venchiarutti Moniz reports a relationship with Amgen Inc that includes: funding grants. Renata Colombo Bonadio reports a relationship with Daiichi Sankyo Inc that includes: consulting or advisory and speaking and lecture fees. Renata Colombo Bonadio reports a relationship with Nestlé Health Science that includes: consulting or advisory and speaking and lecture fees. Renata Colombo Bonadio reports a relationship with Addium that includes: consulting or advisory and speaking and lecture fees. Renata Colombo Bonadio reports a relationship with Gilead that includes: consulting or advisory and speaking and lecture fees. Renata Colombo Bonadio reports a relationship with Merck Sharp & Dohme Corp that includes: consulting or advisory and speaking and lecture fees. Renata Colombo Bonadio reports a relationship with Bristol Myers Squibb Co that includes: consulting or advisory and speaking and lecture fees. Renata Colombo Bonadio reports a relationship with AstraZeneca that includes: consulting or advisory, funding grants, and speaking and lecture fees. Renata Colombo Bonadio reports a relationship with Aché that includes: consulting or advisory and speaking and lecture fees. Renata Colombo Bonadio reports a relationship with Pfizer that includes: consulting or advisory and speaking and lecture fees. Renata Colombo Bonadio reports a relationship with Novartis that includes: consulting or advisory, funding grants, and speaking and lecture fees. Renata Colombo Bonadio reports a relationship with Libbs that includes: consulting or advisory and speaking and lecture fees. Renata Colombo Bonadio reports a relationship with Lilly that includes: consulting or advisory and speaking and lecture fees. Renata Colombo Bonadio reports a relationship with Roche that includes: consulting or advisory and speaking and lecture fees. If there are other authors, they declare that they have no known competing financial interests or personal relationships that could have appeared to influence the work reported in this paper.

## Data Availability

Data available upon request.

## Appendix Supplementary materials

Image, application 1
