# Supplementary material for: Role of dynamic ctDNA monitoring in cervical and anal epidermoid carcinomas under curative chemoradiation
Source: Clinics (Sao Paulo). 2026 Jul 14;81:101048. doi: 10.1016/j.clinsp.2026.101048 (PMC13382435; doi:10.1016/j.clinsp.2026.101048)
Supplement: Supplementary file 1 [file mmc1.docx]

**CLINICS-D-26-00483_ Supplementary Material**

**Supplementary Table 1** ctDNA levels for each patient at each time point during and after treatment with chemoradiation for cervical or anal cancer.


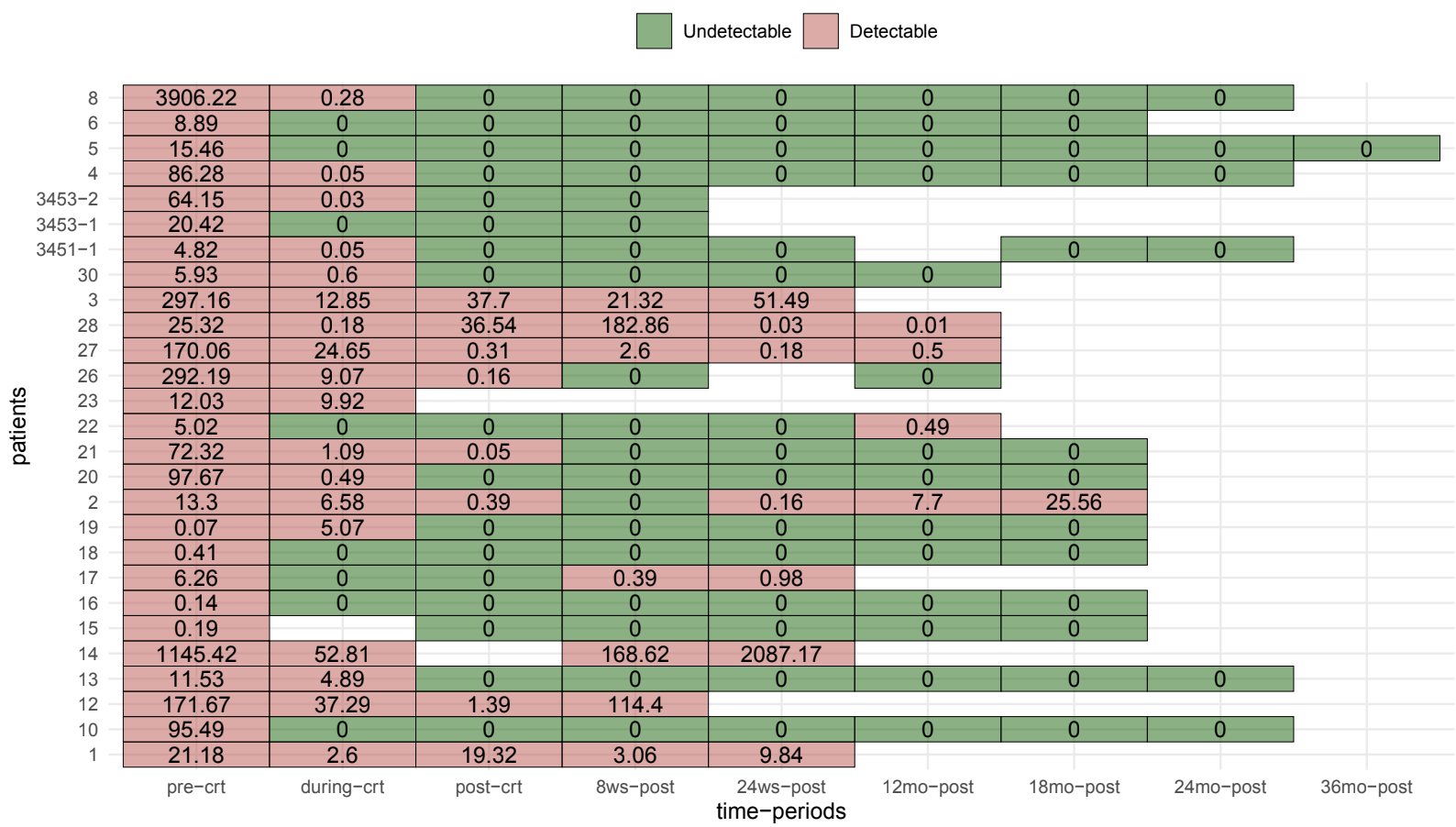


**Supplementary Table 2** Diagnostic performance of 8-week ctDNA and 6-month radiologic response as predictors of disease recurrence in anal cancer (A) and cervical cancer (B) after definitive chemoradiation.

| **A. Anal cancer** | | | | | |
| --- | --- | --- | --- | --- | --- |
| **Test** | **Sensitivity** | **Specificity** | **PPV** | **NPV** | **Accuracy** |
| 8-week ctDNA, % (95% CI) | 66.7% (30–90%) | 100% (70–100%) | 100% (51–100%) | 81.8% (52–95%) | 86.7% (62–96%) |
| 8- to 12-week radiologic response, % (95% CI) | 83.3% (44–97%) | 37.5% (14–69%) | 50% (24–76%) | 75% (30–95%) | 57.1% (33–79%) |
| 6-month radiologic response % (95% CI) | 100% (51–100%) | 33.3% (10–70%) | 66.7% (30–90%) | 100% (51–100%) | 80% (49–94%) |
| **B. Cervical cancer** | | | | | |
| **Test** | **Sensitivity** | **Specificity** | **PPV** | **NPV** | **Accuracy** |
| 8-week ctDNA, % (95% CI) | 75% (30–95%) | 100% (65–100%) | 100% (44–100%) | 87.5% (53–98%) | 90.9% (62–98%) |
| 8- to 12-week radiologic response, % (95% CI) | 100% (51–100%) | 80% (38–96%) | 80% (38–96%) | 100% (51–100%) | 88.9% (56–98%) |
| 6-month radiologic response, % (95% CI) | 100% (51–100%) | 42.8% (16–75%) | 50% (22–78%) | 100% (44–100%) | 63.6% (35–85%) |

**Supplementary Figure 1** Study design and timepoints of ctDNA evaluation.


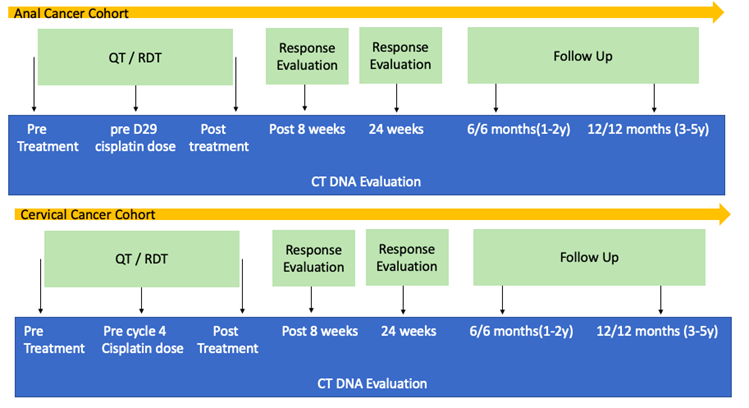


**Supplementary Figure 2** Progression-free survival of patients with anal cancer (A) and cervical cancer (B) cancer after definitive chemoradiation according to 8-week ctDNA status. 8w, 8-weeks; ctDNA+, Detectable ctDNA; ctDNA-, Undetectable ctDNA.
